# SCAPS-1D Modeling of Hydrogenated Lead-Free Cs_2_AgBiBr_6_ Double Perovskite Solar Cells with a Remarkable Efficiency of 26.3%

**DOI:** 10.3390/nano14010048

**Published:** 2023-12-23

**Authors:** Hussein Sabbah, Zaher Abdel Baki, Rabih Mezher, Jack Arayro

**Affiliations:** College of Engineering and Technology, American University of the Middle East, Egaila 54200, Kuwait; zaher.abdelbaki@aum.edu.kw (Z.A.B.); rabih.mezher@aum.edu.kw (R.M.); jack.arayro@aum.edu.kw (J.A.)

**Keywords:** solar cell, photovoltaics, double perovskite, SCAPS simulation, lead-free perovskite, power conversion efficiency

## Abstract

In this investigation, we employ a numerical simulation approach to model a hydrogenated lead-free ${Cs}_{2}AgBi{Br}_{6}$ double perovskite solar cell with a p-i-n inverted structure, utilizing SCAPS-1D. Contrary to traditional lead-based perovskite solar cells, the ${Cs}_{2}AgBi{Br}_{6}$ double perovskite exhibits reduced toxicity and enhanced stability, boasting a maximum power conversion efficiency of 6.37%. Given its potential for improved environmental compatibility, achieving higher efficiency is imperative for its practical implementation in solar cells. This paper offers a comprehensive quantitative analysis of the hydrogenated lead-free ${Cs}_{2}AgBi{Br}_{6}$ double perovskite solar cell, aiming to optimize its structural parameters. Our exploration involves an in-depth investigation of various electron transport layer materials to augment efficiency. Variables that affect the photovoltaic efficiency of the perovskite solar cell are closely examined, including the absorber layer’s thickness and doping concentration, the hole transport layer, and the absorber defect density. We also investigate the impact of the doping concentration of the electron transport layer and the energy level alignment between the absorber and the interface on the photovoltaic output of the cell. After careful consideration, zinc oxide is chosen to serve as the electron transport layer. This optimized configuration surpasses the original structure by over four times, resulting in an impressive power conversion efficiency of 26.3%, an open-circuit voltage of 1.278 V, a fill factor of 88.21%, and a short-circuit current density of $23.30\;mA.{cm}^{-2}$. This study highlights the critical role that numerical simulations play in improving the chances of commercializing Cs_2_AgBiBr_6_ double perovskite solar cells through increased structural optimization and efficiency.

## 1. Introduction

Perovskite solar cells (PSCs) stand out as a major breakthrough in third-generation solar cells, boasting a remarkable 25.7% [1,2,3,4] photoelectronic conversion efficiency (PCE) comparable to silicon-based counterparts. This achievement highlights their potential as a viable alternative in solar energy research. The notable increase in PCE can be attributed to perovskite’s exceptional optical and photophysical properties [5,6,7,8], as well as the collaborative efforts in optimizing materials, refining device architecture, and enhancing interfacial engineering techniques [9,10,11,12,13]. Perovskite exhibits outstanding characteristics, including broad-spectrum absorption ranging from visible to near-infrared wavelengths [14] and a high extinction coefficient, ensuring saturated light absorption within a thickness of 400–500 nm [15]. Additionally, its low exciton binding energy, leading to dissociation at room temperature, long diffusion lengths, and high tolerance for defects, all contribute to its advantages in photovoltaic technology [16,17,18,19]. Despite their enormous potential, the presence of toxic lead (Pb) in the B-site structure is essential for achieving high efficiencies, as per the ${ABX}_{3}$ crystal arrangement, which includes organic/inorganic monovalent cations (A), a divalent cation (B), and one or more halides (X). However, hurdles such as current−voltage hysteresis, limited stability, lead toxicity, and inadequate water resistance continue to impede the widespread adoption of these lead-based PSCs [20,21].

Several low-toxicity metal halide options have been proposed as substitutes for lead in prior research [22,23,24,25]. However, owing to its favorable physical and optical properties, tin (Sn) has emerged as the most promising alternative [26,27]. Tin is preferred for lead-free PSCs because of its comparable isoelectronic configuration ($s^{2}p^{2}$) and superior mobility when compared to lead-based counterparts [28]. Additionally, tin-based perovskites exhibit an optical bandgap ranging from 1.2 to 1.4 eV [29], closely aligning with the ideal bandgap of 1.34 eV, as per the Shockley−Queisser limit under AM 1.5 solar spectrum illumination [30]. Recent advancements have showcased tin-based perovskites achieving a remarkable record PCE of over 13% [31], coupled with excellent device stability [31,32], making them promising substitutes for lead-based perovskites. However, a notable drawback arises from the susceptibility of ${Sn}^{2+}$ ions to oxidation, transitioning into ${Sn}^{4+}$ ions in the presence of air due to poor stability [33]. This phenomenon results in the degradation of photovoltaic performance.

The persistent quest for lead-free perovskites with robust inherent stability in solar cell technology is a vital yet challenging pursuit. One promising avenue involves creating a lead-free active layer while retaining the fundamental perovskite crystal structure. This is achieved by replacing two ${Pb}^{2+}$ cations with non-toxic heterovalent metal cations, each having oxidation states of +1 and +3. The resulting compound, known as “lead-free double perovskite LFDP”, adopts the $A_{2}{M(I)}^{+}{M(III)}^{3+}X_{6}$ structure, referred to as elpasolite [15]. This family encompasses over 350 different elpasolites [34], showcasing the versatility of LFDPs.

Utilizing first-principles calculations, researchers have pinpointed eleven materials with suitable bandgaps to serve as photovoltaic absorbers [35,36]. However, only a few of these materials have been successfully synthesized, including ${Cs}_{2}AgBi{Br}_{6}$ [37], ${Cs}_{2}AgBi{Cl}_{6}$ [38], and ${\left({CH}_{3}{NH}_{3}\right)}_{2}AgBi{Br}_{6}$ [35]. In these perovskites, the substitution of ${Pb}^{2+}$ with B-site cations like ${Ag}^{+}$ and ${Bi}^{3+}$ significantly enhances stability due to an augmented Coulomb interaction energy [39]. This enhancement results in a remarkably high positive decomposition energy, particularly in ${Cs}_{2}AgBi{Br}_{6}$ (0.38 eV) [39]. Consequently, ${Cs}_{2}AgBi{Br}_{6}$-based PSCs emerge as highly promising contenders within the realm of inorganic lead-free perovskite photovoltaic devices. The reported efficiencies of ${Cs}_{2}AgBi{Br}_{6}$-based PSCs have remained low due to inherent limitations such as large charge carrier effective masses, a significant indirect bandgap, and weak charge carrier transport capabilities. Despite numerous efforts to enhance the optoelectronic properties of ${Cs}_{2}AgBi{Br}_{6}$ PSCs, progress has been slow, with the highest efficiency reaching only 4.23% [40,41,42,43], considerably lower than organic–inorganic hybrid lead-based PSCs. However, recent research by Z. Zhang et al. [44] presented a promising solution. They utilized a hydrogenation method to adjust the ${Cs}_{2}AgBi{Br}_{6}$ films’ bandgap from 2.18 eV to 1.64 eV. This adjustment significantly improved the photoelectric conversion efficiency to 6.37% while maintaining excellent environmental stability. Further investigations revealed that introducing atomic hydrogen into the ${Cs}_{2}AgBi{Br}_{6}$ lattice not only fine-tuned its valence and conduction band energy levels but also enhanced carrier mobility and lifetime. This innovative approach offers a potential solution to the limitations of ${Cs}_{2}AgBi{Br}_{6}$-based PSCs, opening avenues for more efficient and stable solar cell technologies. 

Along with experiments, simulation is integral to understanding the properties and performance metrics of various materials, complementing experimental studies [45,46,47]. Device simulation offers a robust method for enhancing the efficiency of LFDP solar cells after optimizing physical parameters. Recent research employing numerical simulations through various software, notably wXAMPS [48] and SCAPS-1D [49], revealed a peak simulated PCE of 11.69% for LFDP solar cells [50,51].

Our study delves into unexplored territory, offering a comprehensive analysis of the factors influencing the efficiency of hydrogenated ${Cs}_{2}AgBi{Br}_{6}$-based PSCs. Notably, our work stands as the inaugural attempt to simulate these cells, building directly upon the pioneering experimental research conducted by Z. Zhang et al. Their groundbreaking work achieved the highest experimental PCE of 6.37% using a hydrogenated device. In our simulation, we meticulously maintained the inverted (p-i-n) structure designed by Z. Zhang et al., keeping the charge transport layers intact: ${SnO}_{2}$ as the electron transport layer (ETL) and Spiro-OmeTAD as the hole transport layer (HTL). Using one-dimensional device simulation with SCAPS (ver. 3.8) under AM1.5G illumination, we meticulously examined crucial factors such as absorber thickness, doping concentration, and defect density. Additionally, we delved into the influence of the doping concentration and thickness of the HTL on the device’s photovoltaic performance. Furthermore, we explored the impact of band alignment at the interface between the double perovskite and ETL, comparing cells using three different ETL materials: tin (IV) oxide ($SnO_{2}$), zinc oxide (ZnO), and titanium dioxide $({TiO}_{2}$). ZnO and ${TiO}_{2}$ are popular ETLs in PSCs due to their high electron mobility, good energy level alignment with perovskite materials, chemical stability, scalability, tunable properties, and wide bandgap, allowing efficient charge transport, stability, and low-cost production at a large scale [52,53].

Notably, our simulations unveiled the potential of hydrogenated ${Cs}_{2}AgBi{Br}_{6}$-based PSCs with an inverted structure (p-i-n). Through these simulations, we demonstrated that the proposed device could achieve an impressive simulated PCE of nearly 26% and 20% with ZnO and ${SnO}_{2}$ ETL, respectively. 

## 2. Materials and Methods

Our investigation in this study was inspired by the groundbreaking work performed by Z. Zhang et al. [44], who developed the hydrogenated ${Cs}_{2}AgBi{Br}_{6}$ perovskite as a light-absorbing substance and achieved a PCE of 6.37%. We used numerical simulations with SCAPS 3.8, a specialized 1D solar cell modeling program developed at the Department of Electronics and Information Systems of the University of Gent, Belgium [49], while Z. Zhang and colleagues carried out physical experiments. SCAPS specializes in simulating multi-layered solar cells and can support up to seven levels. We carefully computed essential electrical properties within this simulation framework, covering parameters such as PCE, energy band structures at heterojunctions, open-circuit voltage ${(V}_{oc})$, short-circuit current density ${(J}_{sc})$, quantum efficiency QE, current density distribution, and fill factor (FF). Solving customized algorithms was necessary for these simulations. To solve these algorithms, SCAPS uses a unique approach that focuses on Poisson’s equation (Equation (1)) as well as the continuity equations for both electrons and holes, which are described in Equations (2) and (3), respectively.

(1) $$\frac{d}{dx}\left(-\varepsilon \left(x\right)\frac{d\psi }{dx}\right)=q\left[p\left(x\right)-n\left(x\right)+N_{D}^{+}\left(x\right)-N_{A}^{-}\left(x\right)+p_{t}\left(x\right)-n_{t}\left(x\right)\right]$$

(2) $$\frac{dp_{n}}{dt}=G_{p}-\frac{p_{n}-p_{n0}}{\tau_{p}}+p_{n}\mu_{p}\frac{d\xi }{dx}+\mu_{p}\xi \frac{dp_{n}}{dx}+D_{p}\frac{d^{2}p_{n}}{{dx}^{2}}$$

(3) $$\frac{dn_{p}}{dt}=G_{n}-\frac{n_{p}-n_{p0}}{\tau_{n}}+n_{p}\mu_{n}\frac{d\xi }{dx}+\mu_{n}\xi \frac{dn_{p}}{dx}+D_{n}\frac{d^{2}n_{p}}{{dx}^{2}}$$

**Figure 1:**
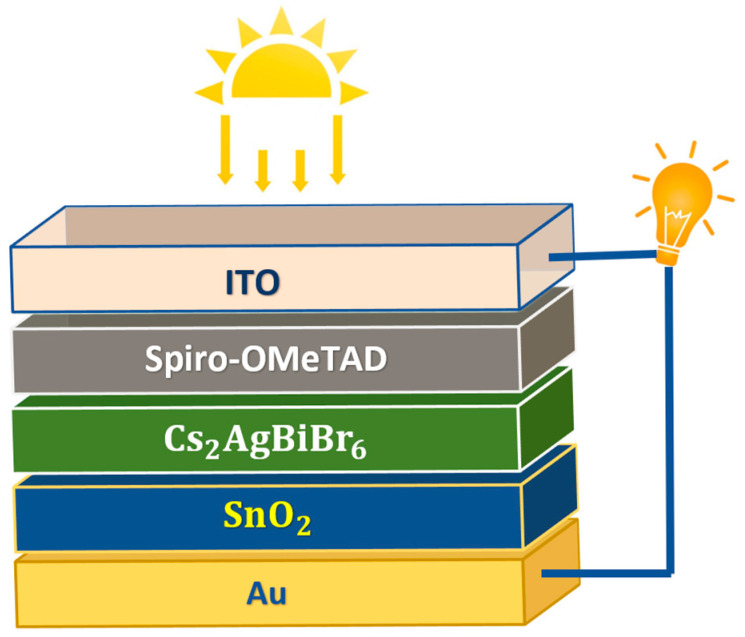
Schematic diagram of ${Cs}_{2}AgBi{Br}_{6}$-based PSC.

Under standard conditions, including a temperature of 300 K, irradiation intensity of $1000\;W/m^{2}$, and air mass AM 1.5 G, our computations positioned the absorber layer between the HTL and the ETL. The proposed structure for the LFDP solar cell is illustrated in Figure 1, featuring gold (Au) as the back contact and indium tin oxide (ITO) as the front contact. 

This configuration follows an inverted (p-i-n) arrangement, where light penetrates the cell from the HTL side. The initial cell design, employing ${Cs}_{2}AgBi{Br}_{6}$ as the absorber material, Spiro-OMeTAD as the HTL, and ${SnO}_{2}$ as the ETL, was borrowed from Z. Zhang et al.’s experimental research. Table 1 and Table 2 outline the electrical and optical properties of these materials [11,15,30,37,38,39,40].

**Figure 2:**
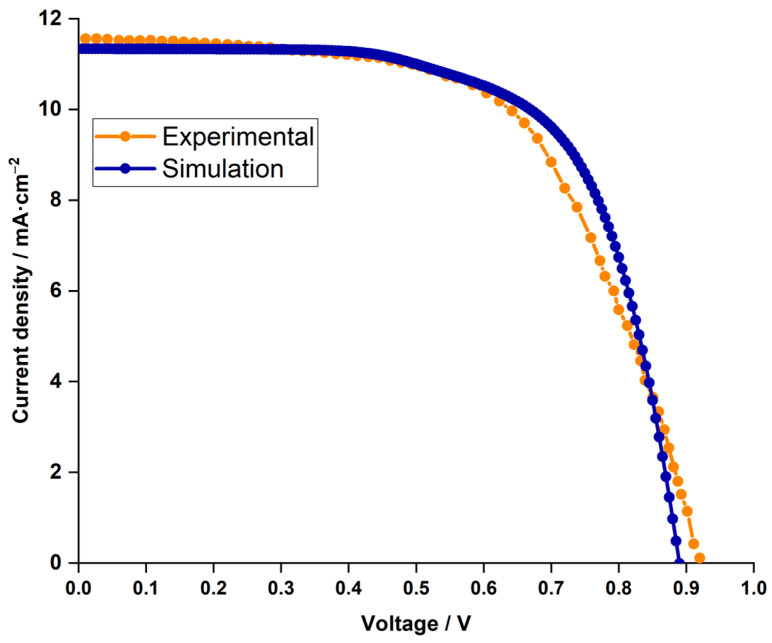
Comparison of current density (J–V curve) between simulation results (depicted in dark blue) and experimental findings (illustrated in orange).

We first compared the current density–voltage characteristics (J–V curve) between the results of our SCAPS simulation and the experimental data to validate our simulations [30]. Interestingly, as seen in Figure 2, the curves had almost perfect overlap, demonstrating the accuracy and dependability of our computational strategy. Critical performance characteristics for the cell were provided by the experimental study, including $J_{sc}$ of $11.36\;mA.{cm}^{-2}$ mA.cm, $J_{sc}$ of 0.89 V, FF of 55.57%, and PCE of 6.37%.

In our research, Spiro-OMeTAD remained the constant choice as the HTL in all our experimental setups. To comprehensively gauge its impact on the device’s performance, we intentionally manipulated several critical parameters. These included not only the thickness of the absorber, defect density, and doping concentration but also the thickness and doping concentration of the HTL itself. Moreover, we systematically varied the electron affinity of the ETL to meticulously assess its influence on band alignment and, consequently, the photovoltaic output of the device. In conclusion, as an alternative to ${SnO}_{2}$, ${TiO}_{2}$ and ZnO have been proposed due to their superior band alignment with the absorber. We quantitatively compared the solar cell devices with three different ETL materials to assess their performance. Our primary goal through these meticulous adjustments was to significantly enhance cell efficiency by intricately refining the cell’s overall structure.

## 3. Results and Discussion

In this section, we explore the study’s outcomes. We optimized the absorber layer by adjusting its thickness, doping concentration, and defect density, identifying the ideal parameters for LFDP solar cells. Subsequently, we fine-tuned the HTL thickness and doping concentration to boost device performance. Additionally, we investigated band alignment’s impact on solar cell efficiency. By proposing alternative ETLs and conducting a detailed analysis, we highlighted the superior performance of ZnO ETL, especially in different doping concentrations. This study provides crucial insights into enhancing hydrogenated double perovskite solar cells, guiding advancements in photovoltaic technology.

### 3.1. Influence of the LFDP Layer on Solar Cell Efficiency

Optimizing the absorber layer in inverted structure perovskite solar cells through precise adjustments in thickness, doping concentration, and defect density is paramount for enhancing their overall efficiency and performance. A well-optimized absorber layer ensures maximized utilization of incident sunlight, leading to improved energy conversion efficiency and the potential for more sustainable and cost-effective solar energy solutions. 

#### 3.1.1. Impact of the LFDP Thickness

The performance of perovskite solar cells is heavily influenced by the thickness of the absorber layer, which directly affects the extent of light absorption and the efficiency of the conversion process. Maintaining an ideal thickness range is essential; if the layer is too thin, it might not absorb adequate light to produce sufficient current. On the contrary, an excessively thick absorber layer could impede the movement of charge carriers generated by absorbed light, making it challenging for them to travel through the material and reach the electrodes. This, in turn, results in reduced device efficiency. Striking the right balance in absorber layer thickness is critical for optimal solar cell performance.

**Figure 3:**
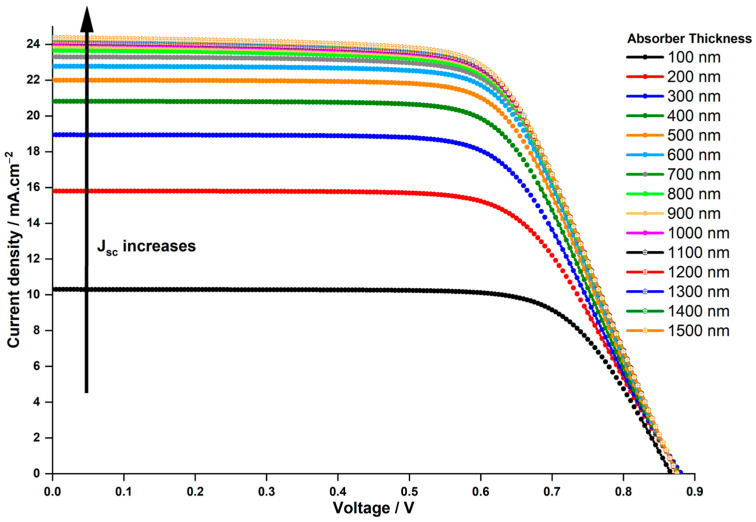
Impact of absorber thickness on J–V characteristics of ${Cs}_{2}AgBi{Br}_{6}$-based PSC.

**Figure 4:**
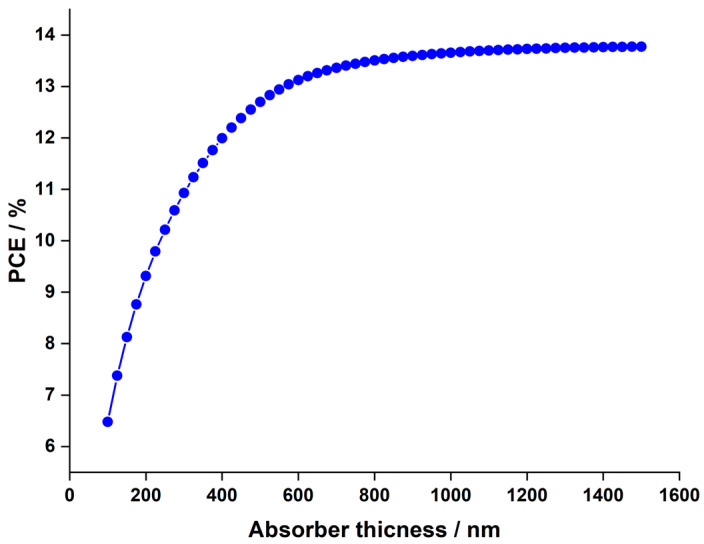
Change in  PCE as function of absorber thickness in ${Cs}_{2}AgBi{Br}_{6}$-based PSC.

In the experimental analyses, a hydrogenated ${Cs}_{2}AgBi{Br}_{6}$ layer with a thickness of 140 nm was utilized [44]. This section investigates the influence of absorber thickness on solar cell performance by adjusting the thickness within the range of 100 nm to 1500 nm. Figure 3 illustrates the J−V characteristics for devices with thickness ranging from 100 nm to 1500 nm, and Figure 4 depicts the PCE in relation to the thickness variations.

**Figure 5:**
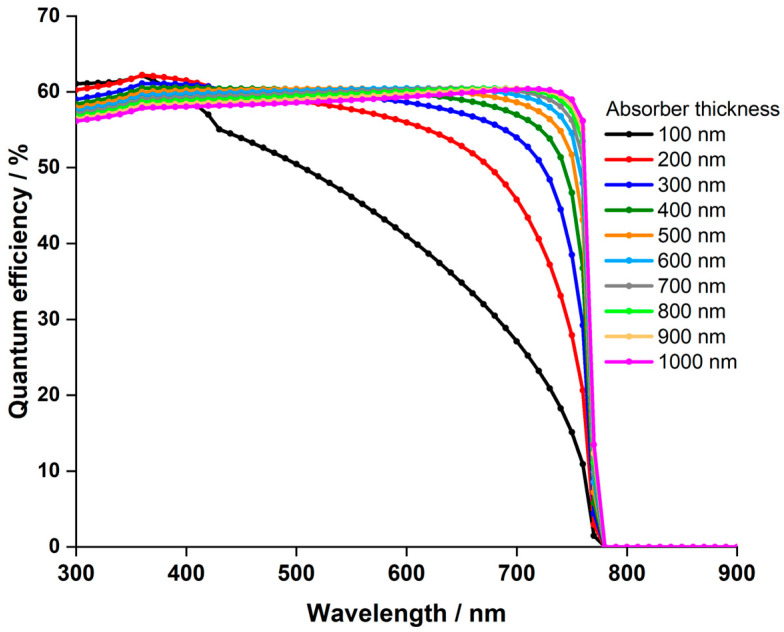
The effect of absorber thickness on the quantum efficiency of ${Cs}_{2}AgBi{Br}_{6}$-based PSC.

The variation in $V_{oc}$ remains minimal and is independent of the thickness. However, $J_{sc}$ experiences a significant increase, rising from 10.29 to $23.31\;mA.{cm}^{-2}$ as the thickness escalates from 100 to 700 nm. Consequently, this leads to a parallel rise in the PCE, escalating from 6.47% to 13.5%. Beyond 700 nm up to 1500 nm, $J_{sc}$ marginally increases, reaching $24.32\;mA.{cm}^{-2}$, while PCE only inches up to 13.75%, a mere 0.25% higher than the PCE at 700 nm, indicating a saturation point in PCE. The behavior described can be thoroughly analyzed through the external quantum efficiency curve of the cell at different thicknesses as a function of incident light wavelengths, depicted in Figure 5. The observed increasing trends in $J_{sc}$ and PCE in Figure 3 and Figure 4, respectively, as the absorber thickness increases up to 700 nm can be explained by enhanced light absorption. This aligns with the peak of quantum efficiency at this thickness in Figure 5.

Moreover, in Figure 5, it is apparent that quantum efficiency declines at wavelengths below 600 nm when the absorber thickness exceeds 700 nm, while it rises for longer wavelengths. Our study’s results match those of S. Dipta et al. [62], although they focused on a lead-based ${CH}_{3}{NH}_{3}{PbI}_{3}$ perovskite in their research. This pattern clarifies the saturated values of $J_{sc}$ and PCE observed for absorber thicknesses surpassing 700 nm. The high absorption coefficient of perovskite material at short wavelengths [63,64] leads to reduced quantum efficiency with thicker layers due to amplified light absorption. Thicker layers intensify the likelihood of absorbed photons generating electron-hole pairs, yet they may also elevate recombination events, wherein electron-hole pairs recombine before reaching the electrodes. This recombination diminishes quantum efficiency, especially at higher photon energies associated with shorter wavelengths.

Conversely, at longer wavelengths, the augmented quantum efficiency with increased thickness can be attributed to multiple factors. Thicker layers bolster the likelihood of light absorption, enabling more photons to be absorbed and creating electron-hole pairs. Moreover, at lower photon energies of longer wavelengths, recombination events are less probable. Hence, thicker layers enhance quantum efficiency, as a larger portion of absorbed photons contributes to charge carrier generation, minimizing losses from recombination processes.

In summary, the behavior of quantum efficiency in LFDP materials is shaped by the complex interactions among light absorption, recombination rates, and photon energy at various wavelengths and thicknesses. Based on these thorough findings, a thickness of 700 nm is chosen for the duration of this investigation.

#### 3.1.2. Impact of Doping Concentration $N_{A}$ and Defect Density $N_{t}$ in the Absorber Layer

Choosing an appropriate absorber thickness is vital in solar cell design, but the defect density $N_{t}$ and acceptor doping concentration $N_{A}$ also play crucial roles. A higher defect density $N_{t}$ leads to increased defects, raising carrier recombination rates and negatively impacting device output [65,66]. Conversely, an increase in acceptor doping concentration $N_{A}$ has been correlated with improved overall solar cell performance [60].

**Figure 6:**
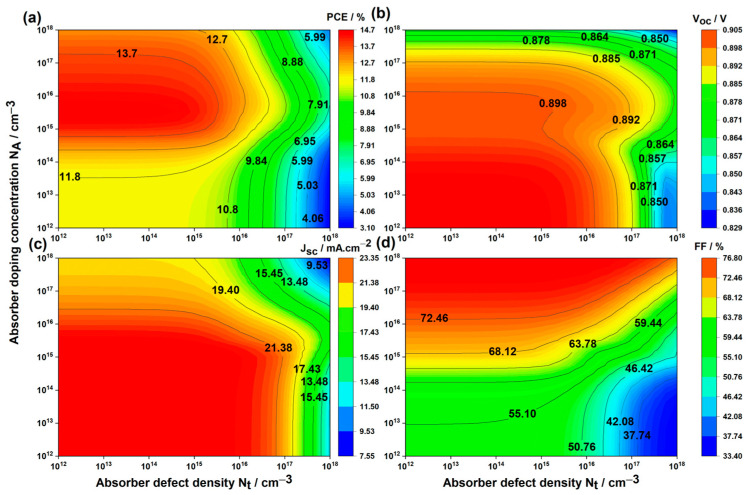
Variations in solar cell performance based on absorber defect densities and absorber doping concentration: (**a**) PCE, (**b**) $V_{oc}$, (**c**) $J_{sc}$, and (**d**) FF.

In Figure 6, the variation of key parameters (PCE, $V_{oc}$, $J_{sc}$, and FF) in the ${Cs}_{2}AgBi{Br}_{6}$-based PSC is depicted concerning absorber defect density $N_{t}$ (*x*-axis) and absorber doping concentration $N_{A}$ (*y*-axis), ranging from $10^{12}$ to $10^{18}\;{cm}^{-3}$. 

Below ${N_{t}=10}^{16}\;{cm}^{-3}$, regardless of $N_{A}$ values, the PSC’s properties remained stable, but deterioration occurred when the defect density exceeded $10^{16}\;{cm}^{-3}$. Defects in the absorber layer act as recombination centers and trap states, reducing efficiency by limiting the number of carriers contributing to the electric current. These defects shorten carrier lifetimes, destabilize the material, and cause voltage losses. To enhance solar cell efficiency and reliability, minimizing defect density is essential. Studies have demonstrated that fabricating tin-based perovskite devices with defect density as low as ${N_{t}=10}^{15}\;{cm}^{-3}$ is achievable [60], making this the optimized value.

The absorber acceptor doping concentration $N_{A}$ significantly influences the PCE of the solar cell, as calculated using Equation (4), and is proportional to $V_{oc}$, $J_{sc}$, and FF.

(4) $$PCE=\frac{V_{oc}\times J_{sc}\times FF}{P_{in}},$$

where $P_{in}$ is the incident power density in watts per square meter.

Analysis of Figure 6a indicates that the maximum PCE values are achieved within the doping concentration range of $10^{12}$ to $10^{18}\;{cm}^{-3}$. This is attributed to the contradictory behavior of $V_{oc}$ and $J_{sc}$ on one hand, and FF on the other, concerning the doping concentration. Observations from Figure 6b,c reveal that $V_{oc}$ and $J_{sc}$ remain constant until $N_{A}$ reaches $10^{16}\;{cm}^{-3}$, after which they decrease. Contrarily, FF (Figure 6d) increases with higher doping concentrations. Our findings about how $N_{A}$ affects LFDP solar cell output perfectly match what Hui-Jing Du et al. discovered while studying lead-free ${CH}_{3}{NH}_{3}{SnI}_{3}$CH_3_ [60].

Increasing acceptor doping boosts the concentration of free charge carriers, enhancing charge separation efficiency at the absorber/ETL interface. More photogenerated carriers contribute to $V_{oc}$ and $J_{sc}$. However, as acceptor doping approaches ETL perovskite’s donor doping concentration $N_{D}=10^{18}\;{cm}^{-3}$, charge recombination intensifies, reducing $V_{oc}$ and $J_{sc}$. The proximity of acceptor and donor dopants accelerates charge recombination, diminishing device performance. However, FF, a dimensionless parameter representing the squareness of the current–voltage curve, is positively influenced by doping. Doping enhances absorber layer conductivity, facilitating efficient charge transport to electrodes and lowering series resistance, thereby improving FF. Additionally, doping affects shunt resistance, reducing unwanted current paths and leakage currents, further enhancing FF. 

The interplay between enhanced conductivity, FF, and increased charge recombination, leading to decrease in $V_{oc}$ and $J_{sc}$, influences PCE in perovskite solar cells. As acceptor doping concentrations of the absorber and donor doping concentration of the ETL approach each other, PCE decreases. It is crucial to balance enhanced conductivity and FF against charge recombination. The optimized value for ${N_{A}=10}^{16}\;{cm}^{-3}$ leads to a PCE of 14.52%, $V_{oc}$ of 0.898 V, $J_{sc}$ of $23.02\;mA.{cm}^{-2}$, and FF of 71.36%.

### 3.2. Influence of the HTL on LFDP Solar Cell Efficiency

In the context of (p-i-n) inverted structure PSCs, the HTL plays a pivotal role in ensuring the efficiency and stability of the device. It achieves this by enhancing charge extraction, reducing interfacial recombination, and modifying band alignment. Among the various HTL materials, Spiro-OMeTAD stands out due to its unique properties. Spiro-OMeTAD, with a bandgap of 2.9 eV, significantly enhances transparency, thereby minimizing optical losses before light penetrates the active double perovskite layer. Its excellent band alignment with the LFDP, coupled with good hole mobility, stability, ease of processing, and compatibility with flexible substrates, makes Spiro-OMeTAD an optimal choice for researchers and manufacturers dedicated to advancing perovskite solar cell technology. However, despite the remarkable properties of Spiro-OMeTAD, there is still room for improvement in the overall efficiency of PSCs. Specifically, optimization of the HTL thickness and doping concentration presents an avenue for enhancing the performance of these solar cells. These parameters demand meticulous exploration and adjustment to further elevate the efficiency and stability of Spiro-OMeTAD-based PSCs.

#### 3.2.1. Impact of the HTL Thickness

**Figure 7:**
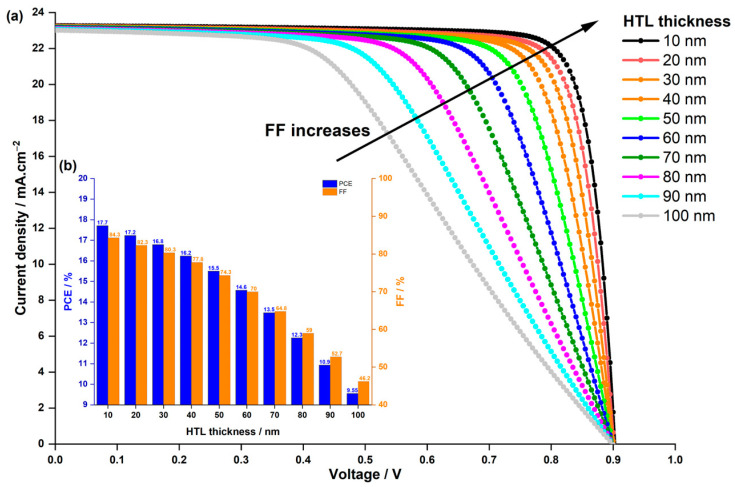
Effect of HTL thickness on (**a**) the current density–voltage characteristics of the PSC, and (**b**) the PCE and FF.

Previous studies have underscored the critical influence of HTL thickness on PSC performance [67,68]. Strikingly, an ultra-thin HTL layer fails to completely cover the absorber layer. Conversely, a thick HTL layer heightens the risk of recombination due to the extended path length of charge carriers and increased electric resistance within the device. Thus, meticulous control of HTL thickness is paramount, aiming for full coverage of the uneven perovskite layer without escalating series resistance in the devices. Consequently, we conducted a study to identify the optimal HTL thickness. We varied the HTL thickness from 10 to 100 nm and scrutinized the photovoltaic performances. The initial thickness of Spiro-OmeTAd in the experimental work was 60 nm [44]. Figure 7 illustrates both the influence of HTL thickness on current density–voltage characteristics and its impact on FF and PCE. As depicted in Figure 7a, $V_{oc}$ remains constant across various thicknesses, while $J_{sc}$ marginally increases from $23.02\;mA.{cm}^{-2}$ to $23.26\;mA.{cm}^{-2}$, ranging from 10 nm to 100 nm thickness. The substantial enhancement is observed in FF, as shown in Figure 7b, and consequently, in PCE, which is directly proportional to $J_{sc}$ and FF. FF elevates from 46.2% to 84.3%, leading to an increase in PCE from 9.55% to 17.7%. This improvement primarily stems from the fact that a thinner layer enhances light transparency, enabling more light to be absorbed by the LFDP layer. Consequently, more carriers are generated, augmenting $J_{sc}$ and FF, which essentially measures the device’s efficacy in converting incident light into electrical current. Additionally, a thinner layer reduces series resistance and improves charge collection efficiency. In a thin HTL, charges have a shorter distance to travel to reach the electrode, minimizing losses and enhancing FF.

#### 3.2.2. Impact of the HTL Doping Concentration $N_{A}$

**Figure 8:**
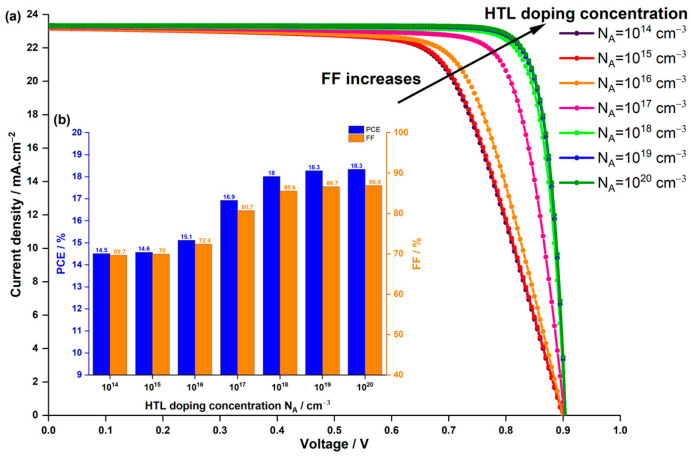
Effect of HTL doping concentration on (**a**) the current density–voltage characteristics of the PSC, and (**b**) the PCE and FF.

Optimizing the performance of HTL involves considering not only the HTL thickness but also the doping concentration $N_{A}$ and its impact on photovoltaic parameters in PSCs. In the experimental study [44], a fixed doping concentration of $N_{A}=1\times 10^{15}\;{cm}^{-3}$ was utilized. However, our numerical analysis explored the effect of varying $N_{A}$ from $1\times 10^{14}$ to $1\times 10^{20}\;{cm}^{-3}$ while keeping the thickness constant at its original value of 60 nm. The results, as depicted in Figure 8, demonstrated comparable trends in photovoltaic metrics with variations in doping concentration, akin to the patterns observed with changes in HTL thickness (Figure 7). 

Notably, for doping concentration, $J_{sc}$, FF, and PCE increased with higher doping concentrations, contrasting the behaviour seen with increasing HTL thickness. Furthermore, it is noteworthy that $V_{oc}$ remained constant in both cases, indicating a consistent $V_{oc}$ across different doping concentrations (Figure 8a) and HTL thicknesses (Figure 7a). Specifically in Figure 8a, $J_{sc}$ marginally rose from $23.16\;mA.{cm}^{-2}$ at $N_{A}=1\times 10^{14}\;{cm}^{-3}$ and 60 nm thickness to $23.26\;mA.{cm}^{-2}$ $23.33\;mA.{cm}^{-2}$ at $N_{A}=1\times 10^{20}\;{cm}^{-3}$ with the same thickness. As depicted in Figure 8b, FF and consequently PCE exhibited a notable increase from 69.5% and 14.65% at $N_{A}=1\times 10^{14}\;{cm}^{-3}$ to 85.6% and 18% at $N_{A}=1\times 10^{18}\;{cm}^{-3}$, after which both metrics approached saturation even with further increases in doping concentration to $N_{A}=1\times 10^{20}\;{cm}^{-3}$. This enhancement in performance can be attributed to HTL doping, which enhances the mobility of charge carriers, particularly holes. Higher carrier mobility enables freer movement of charges within the material, reducing resistive losses and enhancing overall device conductivity. Doping also minimizes recombination by providing additional charge carriers to neutralize traps and defects within the material. Additionally, doping ensures appropriate energy level matching, thereby enhancing charge transfer efficiency.

**Figure 9:**
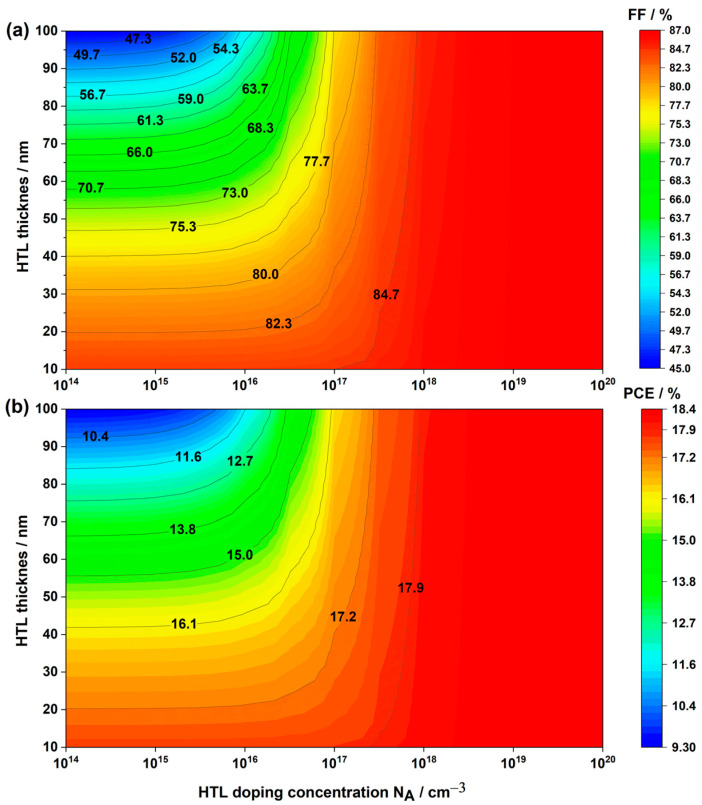
Variations in solar cell performance based on HTL doping concentration and HTL thickness: (**a**) FF and (**b**) PCE.

In our pursuit of optimizing the HTL parameters for ${Cs}_{2}AgBi{Br}_{6}$-based PSC, we delved into the relationship between HTL thickness and HTL doping concentration. Figure 9 illustrates the variations in PCE and FF in PSCs based on ${Cs}_{2}AgBi{Br}_{6}$ concerning HTL doping concentration (ranging from $1\times 10^{14}$ to $1\times 10^{20}\;{cm}^{-3}$) and HTL thickness (ranging from 10 to 100 nm). 

Notably, our findings reveal an inverse correlation between HTL thickness and doping concentration in inverted PSCs. Specifically, PCE and FF increased with higher doping concentration and decreased with thicker HTL thickness. It is noteworthy that an optimal balance exists; going below a thickness of 20 nm does not necessarily enhance cell performance, and an excessively high doping concentration, such as $1\times 10^{20}\;{cm}^{-3}$, is unnecessary. A concentration of $1\times 10^{18}\;{cm}^{-3}$ suffices, especially considering the rising fabrication costs and complexity associated with higher doping levels [69]. Consequently, for the remainder of our study, we adopted an HTL thickness of 20 nm and a doping concentration of $1\times 10^{18}\;{cm}^{-3}$, resulting in impressive photovoltaic parameters: a PCE of 18.15%, $V_{oc}$ of 0.903 V, $J_{sc}$ of $23.27\;mA.{cm}^{-2}$, and FF of 86.56%.

### 3.3. Influence of the ETL on LFDP Solar Cell Efficiency

Careful selection of ETL parameters is paramount in the design of high-performing solar cells, as emphasized in previous research [34]. ETLs within PSCs play vital roles, facilitating the collection and transfer of charge carriers following electron injection from the perovskite active layer. Of utmost significance is their ability to achieve effective charge separation and suppress charge carrier recombination. This intricate process hinges on optimizing factors such as the conduction band offset (CBO) and doping concentration. In this study, we investigate the influence of CBO on solar cell performance. Our analysis involves a comparative examination of the experimental structure used by Zhan et al. against two other cells utilizing the commonly employed metal oxides, titanium dioxide (${TiO}_{2}$) and zinc oxide (ZnO), as ETLs. Notably, we explore the photovoltaic outputs of these three devices, each with varying doping concentrations. Through this exploration, we gain valuable insights into the interplay between ETL parameters and the overall efficiency of PSCs, shedding light on the intricate mechanisms behind their optimal functioning.

#### 3.3.1. Impact of the CBO

Recently, extensive research efforts have been dedicated to finding suitable ETLs for PSCs to enhance charge carrier transport. This pursuit is driven by the fact that the $V_{oc}$ values below 1 V in most lead-free PSCs are insufficient when compared to the typical optical bandgap of the light absorber. The open-circuit losses in PSCs primarily stem from recombination processes within the perovskite bulk layer, as discussed in Section 3.1.2, and at the LFDP/ETL interface. To investigate the impact of the CBO between the LFDP and ETL, the back junction band alignment of the ${Cs}_{2}AgBi{Br}_{6}$-based PSC was examined. CBO can be calculated using Equation (5), and its effect on the current density–voltage characteristics and PCE was explored.

(5) $$CBO=\chi_{Absorber}-\chi_{ETL}$$

**Figure 10:**
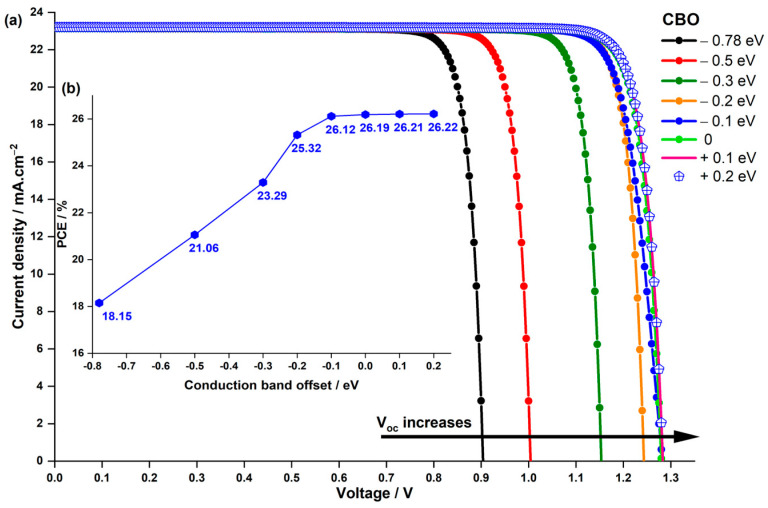
Impact of CBO on (**a**) the current density–voltage characteristics of the PSC and (**b**) the PCE.

In simulations where the ETL’s energy gap was constant at 3.6 eV, the CBO between the LFDP and ETL varied from −0.78 eV to 0.2 eV, achieved by altering the electron affinity $\chi_{ETL}$ from −4.5 eV to −3.52 eV while maintaining a constant electron affinity of the absorber $\chi_{absorber}=3.72\;eV.$ Figure 10 illustrates the results. 

Notably, utilizing experimental values from Z. Zhang et al. [44], when the CBO between the LFDP and ETL was −0.78 eV, the corresponding cell exhibited the lowest $V_{oc}$ value of 0.903 V, resulting in the lowest PCE of 18.15%. However, as the CBO changed from −0.78 to 0 eV, the $V_{oc}$ significantly increased, leading to a higher PCE of 26.19% when $\chi_{ETL}$ was equal to 3.72 eV (Figure 10b). After that, when CBO becomes positive, $V_{oc}$ and PCE saturate. It is also worth mentioning that $J_{sc}$ almost remains the same for all values of CBO.

This enhancement in $V_{oc}$ and consequently in PCE can be attributed to reducing the negative value of CBO. The negative CBO indicates that the energy level of the conduction band in the ETL is lower than that of the perovskite layer. Consequently, a larger energy barrier is created for electrons moving from the perovskite layer to the ETL, decreasing the built-in potential. Additionally, a negative CBO reduces the barrier for electron transport from the ETL’s conduction band to the interface. Since solar cell interfaces often have defect states, particularly deep defects that act as recombination centers for electrons and holes, a negative CBO exacerbates charge recombination through these interface deep-level defects. Given that $V_{oc}$ is essentially the built-in potential minus losses due to recombination and other factors, the increase in recombination at the interfaces due to negative CBO and the decrease in built-in potential resulted in a lower $V_{oc}$ and hence PCE.

**Figure 11:**
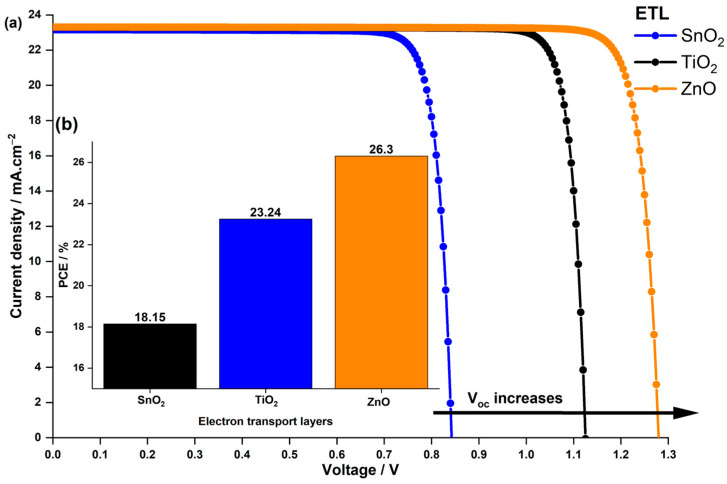
Impact of the ETL material on (**a**) the current density–voltage characteristics of the PSC and (**b**) the PCE.

In real-world applications, finding the right ETL is crucial, and among the promising options are ZnO and ${TiO}_{2}$. ${TiO}_{2}$ has an electron affinity of 4.0 eV, resulting in a CBO of −0.2 eV, while ZnO, with an electron affinity of 3.7 eV, nearly approaches zero  CBO. These values represent significant improvements compared to the −0.78 eV CBO observed between ${SnO}_{2}$ and the absorber material used in the study by Z. Zhang et al. [44]. To assess their performance, we simulated the J−V characteristics and PCE for three different devices with varied ETLs, as depicted in Figure 11. 

The finding suggests that devices incorporating the alternative ${TiO}_{2}$ and ZnO ETLs exhibited superior $V_{oc}$ and PCE compared to the original ${SnO}_{2}$-based device. Notably, ZnO outperformed both ${SnO}_{2}$ and ${TiO}_{2}$ due to its excellent $V_{oc}$ of 1.27 V, resulting in an impressive 26.3% PCE. These results can be attributed to the charge recombination at the interface explained previously, influenced by the CBO effect. Despite comparable electron mobility and conductivity among the proposed ETLs, their distinct alignment with the absorber material played a pivotal role in achieving these outcomes. Apart from the band alignment between the ETL and LFDP layer, their conductivity significantly influences cell design. Consequently, the performance of the optimized cell using ${SnO}_{2}$ as the ETL and the one employing ZnO as the ETL was compared under various doping concentrations.

#### 3.3.2. Impact of the ETL Doping Concentration $N_{D}$

**Figure 12:**
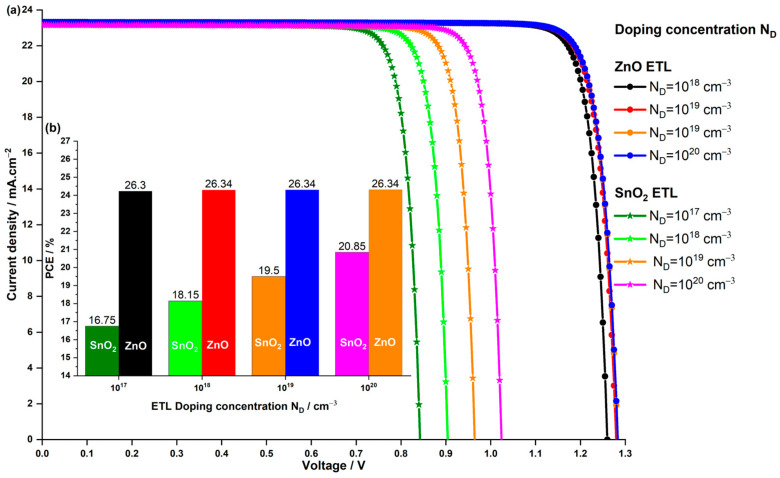
Impact of the doping concentration of the ${SnO}_{2}$ and ZnO ETL on the (**a**) the current density–voltage characteristics of the PSC and (**b**) the PCE.

When dopants are introduced into the ETL, they can alter the charge carrier concentration and the conductivity of the material. This, in turn, affects the built-in electric field, which is crucial for separating and transporting electrons and holes within the solar cell. In our simulations, we varied the shallow donor doping concentration $N_{D}$ of the ETL from ${1\times 10}^{17}$ to ${1\times 10}^{20}\;{cm}^{-3}$. We refrained from going below ${1\times 10}^{17}\;{cm}^{-3}$ based on our findings in Section 3.1.2, where we observed that as the acceptor doping $N_{A}$ in the absorber approaches the donor doping concentration $N_{D}$ of the ETL, charge recombination intensifies, leading to a reduction in both $V_{oc}$ and $J_{sc}$. Figure 12 illustrates the impact of donor doping concentration in both ${SnO}_{2}$ and ZnO ETLs on the J−V characteristics and PCE of the solar cell.

In Figure 12a, it is evident that the $J_{sc}$ experiences a marginal increase with higher ETL doping concentrations for both devices utilizing different ETL materials. The most notable impact is observed in the $V_{oc}$ parameter, particularly in the solar cell employing ${SnO}_{2}$ ETL. For the ZnO ETL-based PSC, $V_{oc}$ rises with increasing ZnO doping concentration from ${1\times 10}^{17}$ to ${1\times 10}^{18}\;{cm}^{-3}$, reaching a saturation point even if $N_{D}$ is increased to ${1\times 10}^{20}\;{cm}^{-3}$. In contrast, the ${SnO}_{2}$ ETL-based device exhibits a substantial effect, with $V_{oc}$ steadily increasing across the doping concentration range, rising from 0.842 V to 1.024 V for ${1\times 10}^{17}$ to ${1\times 10}^{20}\;{cm}^{-3}$, respectively. In Figure 12b, the PCE trend mirrors that of $V_{oc}$. The PCE of the ZnO ETL-based PSC sees a slight increase from 26.30% to 26.34%, while the ${SnO}_{2}$ ETL-based PSC continues to rise, reaching a maximum of 20.85%. Notably, even at the lowest doping concentration of ${1\times 10}^{17}\;{cm}^{-3}$, the PCE of the PSC with ZnO ETL surpasses that of the PSC with the original ${SnO}_{2}$ ETL used in Z. Zhan et al.’s experiment [44]. 

The significant enhancement in the ${SnO}_{2}$ ETL-based PSC’s performance is attributed to the association of increasing doping concentration with improved charge carrier transport properties and reduced recombination losses. As the doping concentration increases, electron mobility within the ${SnO}_{2}$ ETL improves, facilitating easier movement of electrons through the material, thereby reducing resistive losses and enhancing overall charge transport efficiency. Additionally, doping influences the energy levels and band alignment at the interfaces between different layers in the solar cell. Improved band alignment enhances the separation of photo-generated carriers, contributing to a higher open-circuit voltage. However, for the ZnO ETL-based PSC, which boasts six times better electron mobility than ${SnO}_{2}$ (as indicated in Table 1) and excellent band alignment with a CBO almost equal to zero, further increases in doping concentration may not significantly enhance the $V_{oc}$ of the PSC with ZnO ETL. Despite the advancements achieved with the ${SnO}_{2}$ ETL-based PSC, the device with ZnO remains the optimal choice, even at $N_{D}={1\times 10}^{18}\;{cm}^{-3}$, displaying a PCE of 26.34%, $V_{oc}$ of 1.278 V, $J_{sc}$ of $23.30\;mA.{cm}^{-2}$, and FF of 88.21%.

## 4. Conclusions

Although ${Cs}_{2}AgBi{Br}_{6}$-based PSCs have been the subject of ground-breaking investigations, the obtained PCE has continuously dropped below 6.37%, falling short of the necessary threshold for commercial viability. Using SCAPS-1D software, we modeled an inverted (p-i-n) structure and carefully evaluated the performance of different parameter layers and materials for the ETL in our study. More specifically, we adjusted the thicknesses of the absorber and HTL, the doping concentrations of the absorber and HTL, and the absorber defect density. Additionally, we improved the efficiency of the solar cell by examining the impact of band alignment at the absorber/ETL interface and investigating the impacts of ETL doping on the device’s overall performance. We proposed ZnO as an optimal ETL alternative to ${SnO}_{2}$, resulting in remarkable enhancements that culminated in an unprecedented PCE of nearly 26.34%, achieved using a lead-free double perovskite as the absorber layer. Looking forward, future research endeavors should focus on refining the techniques employed in device fabrication. Our innovative findings offer a promising avenue for developing cost-effective, highly efficient, and stable ${Cs}_{2}AgBi{Br}_{6}$-based PSCs. These results underscore the significant potential of double perovskite solar cells for future commercial applications.

## Figures and Tables

**Table 1:** Electrical and optical properties used in simulation of a LFDP based on ${Cs}_{2}AgBi{Br}_{6}$.

Parameters	Spiro-OMeTAD (HTL) [44,54,55]	${Cs}_{2}AgBi{Br}_{6}$ (Absorber) [44,51]	${SnO}_{2}$ (ETL) [44,56,57]	ZnO (ETL) [58,59]	${TiO}_{2}$ (ETL) [60,61]
Thickness (µm)	0.060	0.140	0.050	0.05	0.050
Bandgap $E_{g}$ (eV)	2.9	1.61	3.6	3.3	3.26
Electron affinity χ (eV)	2.2	3.72	4.5	3.7	4
Dielectric permittivity	3	5.8	8	9	32
CB effective density of states $\left({cm}^{-3}\right)$	$2.5\times 10^{19}$	$2\times 10^{18}$	$2.2\times 10^{18}$	$2.2\times 10^{18}$	$2.2\times 10^{18}$
VB effective density of states $\left({cm}^{-3}\right)$	$1.8\times 10^{19}$	$1\times 10^{18}$	$1.8\times 10^{19}$	$1.8\times 10^{19}$	$1.8\times 10^{19}$
Electron mobility $\left({cm}^{2}/V.s\right)$	$2\times 10^{-4}$	9.28	15	100	20
Hole mobility $\left({cm}^{2}/V.s\right)$	$2\times 10^{-4}$	9.28	15	25	10
Donor concentration $N_{D}({cm}^{-3})$	$1\times 10^{7}$	0	$1\times 10^{18}$	$1\times 10^{18}$	$1\times 10^{18}$
Acceptor concentration $N_{A}({cm}^{-3})$	$1\times 10^{15}$	$1\times 10^{15}$	0	0	0

**Table 2:** Density values of defects within cell layers and at the cell interface.

Parameters	ETL	HTL	Absorber	HTL/Absorber	Absorber/ETL
Defect Type	Neutral	Neutral	Neutral	Neutral	Neutral
Capture cross-section for electrons $\sigma_{n}\;({cm}^{-2})$	$1\times 10^{-15}$	$1\times 10^{-15}$	$1\times 10^{-15}$	$1\times 10^{-18}$	$1\times 10^{-15}$
Capture cross-section for hole $\sigma_{p}\;({cm}^{-2})$	$1\times 10^{-15}$	$1\times 10^{-15}$	$1\times 10^{-15}$	$1\times 10^{-16}$	$1\times 10^{-15}$
Energetic distribution	Single	Single	Gaussian	Single	Single
Energy level with respect to $E_{v}$ (above $E_{v}$) (eV)	0.6	0.650	0.6	0.6	0.6
Characteristic energy (eV)	0.1	0.1	0.1	0.1	0.1
Total density $N_{t\;}({cm}^{-3})$	$1\times 10^{15}$	$1\times 10^{15}$	$1\times 10^{16}$	$1\times 10^{12}$	$1\times 10^{12}$

## Data Availability

Data are contained within the article.

## References

[1] Shih Y.-C., Lan Y.-B., Li C.-S., Hsieh H.-C., Wang L., Wu C.-I., Lin K.-F. (2017). Amino-Acid-Induced Preferential Orientation of Perovskite Crystals for Enhancing Interfacial Charge Transfer and Photovoltaic Performance. Small.

[2] Huang H.-H., Shih Y.-C., Wang L., Lin K.-F. (2019). Boosting the Ultra-Stable Unencapsulated Perovskite Solar Cells by Using Montmorillonite/CH_3_NH_3_PbI_3_ Nanocomposite as Photoactive Layer. Energy Environ. Sci..

[3] Yang W.S., Park B.-W., Jung E.H., Jeon N.J., Kim Y.C., Lee D.U., Shin S.S., Seo J., Kim E.K., Noh J.H. (2017). Iodide Management in Formamidinium-Lead-Halide–Based Perovskite Layers for Efficient Solar Cells. Science.

[4] Zhong M., Zeng W., Tang H., Wang L.-X., Liu F.-S., Tang B., Liu Q.-J. (2019). Band Structures, Effective Masses and Exciton Binding Energies of Perovskite Polymorphs of CH_3_NH_3_PbI_3_. Solar Energy.

[5] Wang X., Zhang T., Lou Y., Zhao Y. (2019). All-Inorganic Lead-Free Perovskites for Optoelectronic Applications. Mater. Chem. Front..

[6] Miyata A., Mitioglu A., Plochocka P., Portugall O., Wang J.T.-W., Stranks S.D., Snaith H.J., Nicholas R.J. (2015). Direct Measurement of the Exciton Binding Energy and Effective Masses for Charge Carriers in Organic–Inorganic Tri-Halide Perovskites. Nature Phys..

[7] Huang J., Yuan Y., Shao Y., Yan Y. (2017). Understanding the Physical Properties of Hybrid Perovskites for Photovoltaic Applications. Nat. Rev. Mater..

[8] Jeon N.J., Na H., Jung E.H., Yang T.-Y., Lee Y.G., Kim G., Shin H.-W., Il Seok S., Lee J., Seo J. (2018). A Fluorene-Terminated Hole-Transporting Material for Highly Efficient and Stable Perovskite Solar Cells. Nat. Energy.

[9] Yoo J.J., Seo G., Chua M.R., Park T.G., Lu Y., Rotermund F., Kim Y.-K., Moon C.S., Jeon N.J., Correa-Baena J.-P. (2021). Efficient Perovskite Solar Cells via Improved Carrier Management. Nature.

[10] Jeong J., Kim M., Seo J., Lu H., Ahlawat P., Mishra A., Yang Y., Hope M.A., Eickemeyer F.T., Kim M. (2021). Pseudo-Halide Anion Engineering for α-FAPbI3 Perovskite Solar Cells. Nature.

[11] Macdonald T.J., Batmunkh M., Lin C.-T., Kim J., Tune D.D., Ambroz F., Li X., Xu S., Sol C., Papakonstantinou I. (2019). Origin of Performance Enhancement in TiO_2_-Carbon Nanotube Composite Perovskite Solar Cells. Small Methods.

[12] Saliba M., Correa-Baena J.-P., Wolff C.M., Stolterfoht M., Phung N., Albrecht S., Neher D., Abate A. (2018). How to Make over 20% Efficient Perovskite Solar Cells in Regular (n–i–p) and Inverted (p–i–n) Architectures. Chem. Mater..

[13] Jodlowski A.D., Roldán-Carmona C., Grancini G., Salado M., Ralaiarisoa M., Ahmad S., Koch N., Camacho L., de Miguel G., Nazeeruddin M.K. (2017). Large Guanidinium Cation Mixed with Methylammonium in Lead Iodide Perovskites for 19% Efficient Solar Cells. Nat. Energy.

[14] Ng C.H., Lim H.N., Hayase S., Zainal Z., Huang N.M. (2018). Photovoltaic Performances of Mono- and Mixed-Halide Structures for Perovskite Solar Cell: A Review. Renew. Sustain. Energy Rev..

[15] Kung P.-K., Li M.-H., Lin P.-Y., Jhang J.-Y., Pantaler M., Lupascu D.C., Grancini G., Chen P. (2020). Lead-Free Double Perovskites for Perovskite Solar Cells. Solar RRL.

[16] Xing G., Mathews N., Sun S., Lim S.S., Lam Y.M., Grätzel M., Mhaisalkar S., Sum T.C. (2013). Long-Range Balanced Electron- and Hole-Transport Lengths in Organic-Inorganic CH_3_NH_3_PbI_3_. Science.

[17] Choi H., Jeong J., Kim H.-B., Kim S., Walker B., Kim G.-H., Kim J.Y. (2014). Cesium-Doped Methylammonium Lead Iodide Perovskite Light Absorber for Hybrid Solar Cells. Nano Energy.

[18] Van Le Q., Jang H.W., Kim S.Y. (2018). Recent Advances toward High-Efficiency Halide Perovskite Light-Emitting Diodes: Review and Perspective. Small Methods.

[19] Dou L., Yang Y., You J., Hong Z., Chang W.-H., Li G., Yang Y. (2014). Solution-Processed Hybrid Perovskite Photodetectors with High Detectivity. Nat. Commun..

[20] Xu J., Saklatvala R., Mittal S., Deshmukh S., Procopio A. (2020). Recent Progress of Potentiating Immune Checkpoint Blockade with External Stimuli—An Industry Perspective. Adv. Sci..

[21] Yang Y., You J. (2017). Make Perovskite Solar Cells Stable. Nature.

[22] Kopacic I., Friesenbichler B., Hoefler S.F., Kunert B., Plank H., Rath T., Trimmel G. (2018). Enhanced Performance of Germanium Halide Perovskite Solar Cells through Compositional Engineering. ACS Appl. Energy Mater..

[23] Shao S., Liu J., Portale G., Fang H.-H., Blake G.R., ten Brink G.H., Koster L.J.A., Loi M.A. (2018). Highly Reproducible Sn-Based Hybrid Perovskite Solar Cells with 9% Efficiency. Adv. Energy Mater..

[24] Yang D., Lv J., Zhao X., Xu Q., Fu Y., Zhan Y., Zunger A., Zhang L. (2017). Functionality-Directed Screening of Pb-Free Hybrid Organic–Inorganic Perovskites with Desired Intrinsic Photovoltaic Functionalities. Chem. Mater..

[25] Hao F., Stoumpos C.C., Cao D.H., Chang R.P.H., Kanatzidis M.G. (2014). Lead-Free Solid-State Organic–Inorganic Halide Perovskite Solar Cells. Nat. Photon.

[26] Sabbah H., Baki Z.A. (2023). Device Simulation of Highly Stable and 29% Efficient FA_0.75_MA_0.25_Sn_0.95_Ge_0.05_I_3_-Based Perovskite Solar Cell. Nanomaterials.

[27] Nasti G., Abate A. (2020). Tin Halide Perovskite (ASnX_3_) Solar Cells: A Comprehensive Guide toward the Highest Power Conversion Efficiency. Adv. Energy Mater..

[28] Stoumpos C.C., Malliakas C.D., Kanatzidis M.G. (2013). Semiconducting Tin and Lead Iodide Perovskites with Organic Cations: Phase Transitions, High Mobilities, and Near-Infrared Photoluminescent Properties. Inorg. Chem..

[29] Tong J., Gong J., Hu M., Yadavalli S.K., Dai Z., Zhang F., Xiao C., Hao J., Yang M., Anderson M.A. (2021). High-Performance Methylammonium-Free Ideal-Band-Gap Perovskite Solar Cells. Matter.

[30] Rühle S. (2016). Tabulated Values of the Shockley–Queisser Limit for Single Junction Solar Cells. Solar Energy.

[31] Wang C., Zhang Y., Gu F., Zhao Z., Li H., Jiang H., Bian Z., Liu Z. (2021). Illumination Durability and High-Efficiency Sn-Based Perovskite Solar Cell under Coordinated Control of Phenylhydrazine and Halogen Ions. Matter.

[32] Arayro J., Mezher R., Sabbah H. (2023). Comparative Simulation Study of the Performance of Conventional and Inverted Hybrid Tin-Based Perovskite Solar Cells. Coatings.

[33] Byranvand M.M., Zuo W., Imani R., Pazoki M., Saliba M. (2022). Tin-Based Halide Perovskite Materials: Properties and Applications. Chem. Sci..

[34] Giustino F., Snaith H.J. (2016). Toward Lead-Free Perovskite Solar Cells. ACS Energy Lett..

[35] Wei F., Deng Z., Sun S., Zhang F., Evans D.M., Kieslich G., Tominaka S., Carpenter M.A., Zhang J., Bristowe P.D. (2017). Synthesis and Properties of a Lead-Free Hybrid Double Perovskite: (CH_3_NH_3_)_2_AgBiBr_6_. Chem. Mater..

[36] Creutz S.E., Crites E.N., De Siena M.C., Gamelin D.R. (2018). Colloidal Nanocrystals of Lead-Free Double-Perovskite (Elpasolite) Semiconductors: Synthesis and Anion Exchange To Access New Materials. Nano Lett..

[37] Slavney A.H., Hu T., Lindenberg A.M., Karunadasa H.I. (2016). A Bismuth-Halide Double Perovskite with Long Carrier Recombination Lifetime for Photovoltaic Applications. J. Am. Chem. Soc..

[38] McClure E.T., Ball M.R., Windl W., Woodward P.M. (2016). Cs_2_AgBiX_6_ (X = Br, Cl): New Visible Light Absorbing, Lead-Free Halide Perovskite Semiconductors. Chem. Mater..

[39] Savory C.N., Walsh A., Scanlon D.O. (2016). Can Pb-Free Halide Double Perovskites Support High-Efficiency Solar Cells?. ACS Energy Lett..

[40] Wang M., Zeng P., Bai S., Gu J., Li F., Yang Z., Liu M. (2018). High-Quality Sequential-Vapor-Deposited Cs_2_AgBiBr_6_ Thin Films for Lead-Free Perovskite Solar Cells. Solar RRL.

[41] Wang B., Li N., Yang L., Dall’Agnese C., Jena A.K., Miyasaka T., Wang X.-F. (2021). Organic Dye/Cs_2_AgBiBr_6_ Double Perovskite Heterojunction Solar Cells. J. Am. Chem. Soc..

[42] Gao W., Ran C., Xi J., Jiao B., Zhang W., Wu M., Hou X., Wu Z. (2018). High-Quality Cs_2_AgBiBr_6_ Double Perovskite Film for Lead-Free Inverted Planar Heterojunction Solar Cells with 2.2% Efficiency. ChemPhysChem.

[43] Sirtl M.T., Hooijer R., Armer M., Ebadi F.G., Mohammadi M., Maheu C., Weis A., van Gorkom B.T., Häringer S., Janssen R.A.J. (2022). 2D/3D Hybrid Cs_2_AgBiBr_6_ Double Perovskite Solar Cells: Improved Energy Level Alignment for Higher Contact-Selectivity and Large Open Circuit Voltage. Adv. Energy Mater..

[44] Zhang Z., Sun Q., Lu Y., Lu F., Mu X., Wei S.-H., Sui M. (2022). Hydrogenated Cs_2_AgBiBr_6_ for Significantly Improved Efficiency of Lead-Free Inorganic Double Perovskite Solar Cell. Nat. Commun..

[45] Minemoto T., Murata M. (2015). Theoretical Analysis on Effect of Band Offsets in Perovskite Solar Cells. Sol. Energy Mater. Sol. Cells.

[46] Liu F., Zhu J., Wei J., Li Y., Lv M., Yang S., Zhang B., Yao J., Dai S. (2014). Numerical Simulation: Toward the Design of High-Efficiency Planar Perovskite Solar Cells. Appl. Phys. Lett..

[47] Sabbah H. (2022). Numerical Simulation of 30% Efficient Lead-Free Perovskite CsSnGeI3-Based Solar Cells. Materials.

[48] Liu Y., Heinzel D., Rockett A. A New Solar Cell Simulator: WxAMPS. Proceedings of the 2011 37th IEEE Photovoltaic Specialists Conference.

[49] Burgelman M., Nollet P., Degrave S. (2000). Modelling Polycrystalline Semiconductor Solar Cells. Thin Solid. Films.

[50] Islam T., Jani R., Amin S.M.A., Shorowordi K.M., Nishat S.S., Kabir A., Taufique M.F.N., Chowdhury S., Banerjee S., Ahmed S. (2020). Simulation Studies to Quantify the Impacts of Point Defects: An Investigation of Cs_2_AgBiBr_6_ Perovskite Solar Devices Utilizing ZnO and Cu_2_O as the Charge Transport Layers. Comput. Mater. Sci..

[51] Mohandes A., Moradi M., Nadgaran H. (2021). Numerical Simulation of Inorganic Cs2AgBiBr6 as a Lead-Free Perovskite Using Device Simulation SCAPS-1D. Opt. Quant. Electron..

[52] Liu G., Zhong Y., Mao H., Yang J., Dai R., Hu X., Xing Z., Sheng W., Tan L., Chen Y. (2022). Highly Efficient and Stable ZnO-Based MA-Free Perovskite Solar Cells via Overcoming Interfacial Mismatch and Deprotonation Reaction. Chem. Eng. J..

[53] Raj A., Kumar M., Kumar A., Laref A., Singh K., Sharma S., Anshul A. (2022). Effect of Doping Engineering in TiO_2_ Electron Transport Layer on Photovoltaic Performance of Perovskite Solar Cells. Mater. Lett..

[54] Hosseini S.R., Bahramgour M., Yardani Sefidi P., Tabatabaei Mashayekh A., Moradi A., Delibas N., Hosseini M.G., Niaei A. (2022). Investigating the Effect of Non-Ideal Conditions on the Performance of a Planar CH_3_NH_3_PbI_3_-Based Perovskite Solar Cell through SCAPS-1D Simulation. Heliyon.

[55] Seyed-Talebi S.M., Mahmoudi M., Lee C.-H. (2023). A Comprehensive Study of CsSnI_3_-Based Perovskite Solar Cells with Different Hole Transporting Layers and Back Contacts. Micromachines.

[56] Baena J.P.C., Steier L., Tress W., Saliba M., Neutzner S., Matsui T., Giordano F., Jacobsson T.J., Kandada A.R.S., Zakeeruddin S.M. (2015). Highly Efficient Planar Perovskite Solar Cells through Band Alignment Engineering. Energy Environ. Sci..

[57] Vasheghani Farahani S.K., Veal T.D., Mudd J.J., Scanlon D.O., Watson G.W., Bierwagen O., White M.E., Speck J.S., McConville C.F. (2014). Valence-Band Density of States and Surface Electron Accumulation in Epitaxial SnO_2_ Films. Phys. Rev. B.

[58] Doroody C., Rahman K.S., Rosly H.N., Harif M.N., Haque F., Tiong S.K., Amin N. (2020). Impact of High Resistivity Transparent (HRT) Layer in Cadmium Telluride Solar Cells from Numerical Simulation. J. Renew. Sustain. Energy.

[59] Hoye R.L.Z., Ehrler B., Böhm M.L., Muñoz-Rojas D., Altamimi R.M., Alyamani A.Y., Vaynzof Y., Sadhanala A., Ercolano G., Greenham N.C. (2014). Improved Open-Circuit Voltage in ZnO–PbSe Quantum Dot Solar Cells by Understanding and Reducing Losses Arising from the ZnO Conduction Band Tail. Adv. Energy Mater..

[60] Du H.-J., Wang W.-C., Zhu J.-Z. (2016). Device Simulation of Lead-Free CH_3_NH_3_SnI_3_ Perovskite Solar Cells with High Efficiency. Chin. Phys. B.

[61] Stamate M.D. (2003). On the Dielectric Properties of Dc Magnetron TiO_2_ Thin Films. Appl. Surf. Sci..

[62] Dipta S.S., Uddin A., Conibeer G. (2022). Enhanced Light Management and Optimization of Perovskite Solar Cells Incorporating Wavelength Dependent Reflectance Modeling. Heliyon.

[63] Shaikh M.N., Zafar Q., Papadakis A. (2019). A Study of Electromagnetic Light Propagation in a Perovskite-Based Solar Cell via a Computational Modelling Approach. Bull. Mater. Sci..

[64] Chander N., Khan A.F., Chandrasekhar P.S., Thouti E., Swami S.K., Dutta V., Komarala V.K. (2014). Reduced Ultraviolet Light Induced Degradation and Enhanced Light Harvesting Using YVO4:Eu3+ down-Shifting Nano-Phosphor Layer in Organometal Halide Perovskite Solar Cells. Appl. Phys. Lett..

[65] Haider S.Z., Anwar H., Wang M. (2018). A Comprehensive Device Modelling of Perovskite Solar Cell with Inorganic Copper Iodide as Hole Transport Material. Semicond. Sci. Technol..

[66] Karimi E., Ghorashi S.M.B. (2017). Investigation of the Influence of Different Hole-Transporting Materials on the Performance of Perovskite Solar Cells. Optik.

[67] Sabbah H., Arayro J., Mezher R. (2022). Numerical Simulation and Optimization of Highly Stable and Efficient Lead-Free Perovskite FA1−xCsxSnI3-Based Solar Cells Using SCAPS. Materials.

[68] Arumugam G.M., Karunakaran S.K., Liu C., Zhang C., Guo F., Wu S., Mai Y. (2021). Inorganic Hole Transport Layers in Inverted Perovskite Solar Cells: A Review. Nano Select.

[69] Li S., Cao Y.-L., Li W.-H., Bo Z.-S. (2021). A Brief Review of Hole Transporting Materials Commonly Used in Perovskite Solar Cells. Rare Met..

